# Performance of six screening scores for obstructive sleep apnoea in an African population: findings from the BeSAS cross-sectional study

**DOI:** 10.1016/j.sleepx.2026.100190

**Published:** 2026-05-15

**Authors:** Ablo Prudence Wachinou, Boubacar Djelo Diallo, Terence Totah, Geoffroy Solelhac, Mathieu Berger, Pervenche Fosto, Salmane Amidou, Attanon Arnauld Fiogbe, Pedro Marques-Vidal, Gildas Agodokpessi, Dismand Houinato, Roch Christian Johnson, Pierre-Marie Preux, Raphael Heinzer

**Affiliations:** a Faculty of Health Sciences, University of Abomey-Calavi, Cotonou, Benin; b National Teaching Hospital for Tuberculosis and Pulmonary Diseases, Cotonou, Benin; c Laboratory of Epidemiology of Chronic and Neurologic Diseases, Faculty of Health Sciences, University of Abomey-Calavi, Cotonou, Benin; d Department of Pneumo-phtisiology, University Hospital Ignace Deen, Conakry, Guinea; e Center of Investigation and Research on Sleep (CIRS), University Hospital of Lausanne (CHUV) and University of Lausanne (UNIL), Lausanne, Switzerland; f Department of Medicine, Internal Medicine, University Hospital of Lausanne (CHUV) and University of Lausanne (UNIL), Lausanne, Switzerland; g Interfaculty Center for Environmental Training and Research for Sustainable Development (CIFRED), University of Abomey-Calavi, Cotonou, Benin; h Inserm U1094, IRD UMR270, Univ. Limoges, CHU Limoges, EpiMaCT - Epidemiology of Chronic Diseases in Tropical Zone, Institute of Epidemiology and Tropical Neurology, Omega Health, Limoges, France

**Keywords:** Obstructive sleep apnoea, NoSAS, Polygraphy, Africa

## Abstract

**Background:**

A screening tool for obstructive sleep apnoea (OSA) is useful in low-income countries where it may be difficult to access sleep recordings. The objective of this study was to assess the performance of six screening scores compared with objective sleep recording.

**Methods:**

This analysis is based on the “Benin Sleep and Society” (BeSAS) study, in which respiratory polygraphy (PG) was performed using a type III device and OSA screening questionnaires (STOP, STOP-Bang, Berlin, NOSAS [≥8 and ≥ 5), No-Apnea, GOAL) were administered to participants. PG-defined OSA severity categories were defined according to the apnoea-hypopnoea index (AHI): mild (AHI 5 to <15/h), moderate (AHI 15 to <30/h) or severe (AHI ≥30/h), and these were compared to score findings.

**Results:**

A total of 1810 subjects (mean age 45.4 ± 14.6 years; 64.3% women) were included. For moderate to severe OSA, the area under the receiver operating characteristic curve was greatest for GOAL and No-Apnea (0.70, CI 0.67-0.73; 0.70, CI 0.68-0.73), followed by NoSAS_5_ (0.69, CI 0.65-0.73). The highest sensitivity values were for NoSAS_5_ (0.73, CI 0.67-0.79), No-Apnea (0.72, CI 0.66-0.78), and GOAL (0.69, CI 0.67-0.79), while NoSAS_8_ had the highest specificity (0.91, CI 0.90-0.93), followed by Berlin (0.88, CI 0.87-0.90) and GOAL (0.71, CI 0.69-0.73). All scores performed poorly with respect to the positive predictive value (PPV), which was highest with NoSAS_8_ (0.38, CI 0.31-0.44).

**Conclusion:**

Although overall performance was modest for all instruments, NoSAS_8_ showed the highest PPV among the evaluated scores. These findings suggest that NoSAS_8_ may be a useful option to support OSA risk stratification in resource-constrained settings, but should be interpreted with caution.

## Highlights

• NoSAS8 had highest specificity (91%) for moderate-to-severe OSA.
• NoSAS8 showed highest positive predictive value (38%).
• GOAL and No-Apnea showed highest sensitivity (73% and 72%).
• First validation of multiple OSA scores in an African general population.
• Underscores need for resource-adapted OSA screening in low-income settings.

## Graphical abstract

**Figure:**
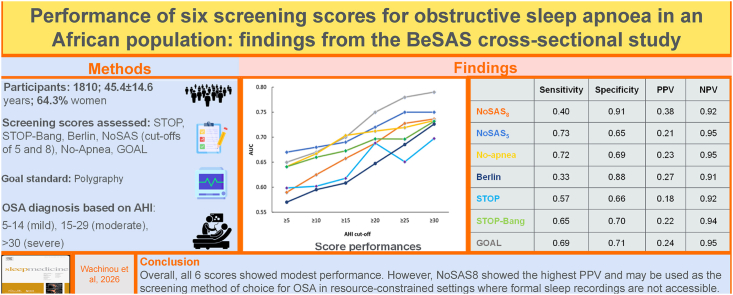

## 1 Introduction

Obstructive sleep apnoea (OSA) is a major public health concern worldwide. Recently published data suggested that up to 425 million individuals aged 30–69 years are affected by at least moderate OSA [1]. Unfortunately, this disease is still underdiagnosed in most parts of the world, especially in low-income countries [2].

OSA is characterised by frequent partial or complete upper airway collapse during sleep, leading to intermittent hypoxemia and sleep fragmentation [3]. The health impacts of OSA include excessive daytime sleepiness, fatigue, road or workplace accidents and, over the long-term, non-communicable diseases (NCDs) such as hypertension, diabetes, depression, cardiovascular disease, neurological conditions, and nonalcoholic fatty liver disease [[4], [5], [6], [7]]. Therefore, there is a link between OSA and most relevant NCDs, which are now among the leading causes of morbidity and mortality worldwide, especially in low and middle-income countries (LMICs) [8].

The central role played by OSA in NCD pathophysiology [9] should prompt early recognition of the condition so that long-term health consequences can be prevented [10]. Unfortunately, polysomnography (PSG), which is the gold standard for OSA diagnosis, and even respiratory polygraphy (PG) as an alternative, are expensive, time-consuming and have limited access, particularly in LMICs, because these procedures require dedicated sleep monitoring facilities and specialised technical staff [11]. Therefore, validated clinical scores are good alternatives to identify individuals at high risk of OSA.

Several screening questionnaires have been developed over the last 30 years, including the Berlin [12], STOP [13], STOP-Bang [14], NoSAS [15], No-Apnea [16] and GOAL [17]. However, little is known about the performance of these questionnaires in LMICs. Indeed, all screening questionnaires have been developed and extensively studied in America, Europe or Asia, but not in Africa. Whether the same defined cut-off points for these questionnaires are applicable in African populations remains unknown. Given the difficulty of accessing sleep laboratory care in LMICs, there is a compelling need to validate an effective OSA screening tool in these populations. In such settings, screening questionnaires are not primarily intended to exclude disease, but rather to identify individuals who are most likely to have clinically relevant OSA and who should therefore be prioritized for limited diagnostic resources or targeted interventions. Consequently, a screening tool with a higher positive predictive value (PPV) is particularly valuable, as PPV reflects the probability that a positive result truly corresponds to disease. Emphasizing PPV in this context may help optimize the allocation of scare diagnostic resources where universal objective sleep testing is not feasible. Thus, the aim of this study was to evaluate the performance of six OSA screening questionnaires in an African general population.

## 2 Methods

### 2.1 Study design

This analysis is based on the cross-sectional Benin Society and Sleep (BeSAS) study conducted in Benin, West Africa, from April 2018 to January 2021. Participants were recruited from both a rural (Tanve village, 52.8%) and an urban (Cotonou, economic capital of Benin, 47.2%) area to best represent the diversity of the Benin population. Detailed description of study recruitment has been published elsewhere [18]. In summary, rural participants were recruited from the Tanve Health Study (TAHES), which is a longitudinal cohort study focused on identifying the incidence of cardiovascular diseases in a rural adult African community. All consenting participants from this cohort were included in the BeSAS study. For urban participants, recruitment took place in the third subcity of Cotonou, the economic capital of Benin. Participants were randomly chosen from 9 out of the 13 districts in the subcity. Households were selected using a proportional sampling technique, and all consenting adults in each selected household were recruited.

The BeSAS study protocol was approved by the National Ethics Committee of Benin (N°45, 25 October 2017) with regular annual approval renewal throughout the course of the research project. Participants were enrolled on a voluntary basis, and all gave written informed consent. If a participant was unable to read, a family member who was able to read was asked to provide study information to the participant to allow informed consent to be obtained.

### 2.2 Demographic and clinical data

Demographic data (age, sex and area of residence), alcohol intake and smoking habits were collected by self-report. Morphometric measurements (height, weight, neck circumference, abdominal circumference) were collected using standardised procedures. Height was measured with a rigid gauge and weight was measured in a standing position, with as little clothing as possible and without shoes, using mechanical devices (Seca, Hamburg, Germany). Nutritional status was assessed using body mass index (BMI), calculated as weight divided by height in metres squared. Normal BMI was defined as <25 kg/m^2^, overweight as BMI 25 to <30 kg/m^2^, and obesity as BMI ≥30 kg/m^2^. Neck and waist circumferences (at the umbilicus) were measured using standardised procedures. Abdominal obesity was defined as a waist circumference ≥102 cm in men and ≥88 cm in women. Blood pressure was measured three times on both arms (Spengler, France) and the average of the last two readings from each arm was calculated. Hypertension was defined as systolic blood pressure ≥140 mm Hg or diastolic blood pressure ≥90 mm Hg on at least one arm, or current use of antihypertensive drugs. A capillary fasting glycaemia test was performed for each subject using a glucometer (Accuchek Performa®, Roche Diagnostics, Basel, Switzerland). Diabetes mellitus was defined as a self-reported medical history of diabetes mellitus or capillary fasting glycaemia ≥7 mmol/L.

Most clinical data were collected during face-to-face interviews using KoBoToolbox software (Harvard Humanitarian Initiative, Cambridge, USA) on digital tablets by trained and experienced interviewers.

### 2.3 OSA screening questionnaires

OSA screening questionnaires were administered to participants by the research assistants using digital tablets. Anthropometric measurements used in all relevant questionnaires were obtained from physical examinations.

1. NoSAS score ranges from 0 to 17 and consists of five items with different relative weight: neck circumference >40 cm: 4 points; BMI <30 kg/m^2^: 3 points and ≥30 kg/m^2^: 5 points; presence of snoring: 2 points; age ≥55 years 4 points; male sex: 2 points. A NoSAS score ≥8 indicates a high risk of OSA [15].
2. No-Apnea score ranges from 0 to 9 and consists of two items: neck circumference scores 0 points if < 37.0 cm, 1 point if 37.0–39.9, 3 points if 40.0–42.9, and 6 points if ≥ 43.0; Age scores 0 points if < 35 years, 1 point if 35–44 years, 2 points if 45–54 years, and 3 points if ≥ 55 years. A No-Apnea score ≥3 indicates a high risk of OSA [16].
3. STOP score ranges from 0 to 4 and consists of four items (snoring, fatigue, observed apnoea and high blood pressure). Each item is answered “yes” or “no” where a “yes” response scores 1 point and a “no” response scores 0 points. A STOP score ≥2 indicates a high risk of OSA [13].
4. STOP-Bang ranges from 0 to 8 and is a STOP score with four other items added: B (BMI >35 kg/m^2^), A (age >50 years), N (neck circumference >40 cm), and G (male sex). Each item is answered with “yes” or “no” where a “yes” response scores 1 point and a “no” response scores 0 points. A STOP-Bang score ≥3 points indicates a high risk of OSA [14].
5. GOAL score ranges from 0 to 4 and consists of four items (male sex, obesity with BMI ≥30 kg/m^2^, age ≥50 years, and loud snoring), with each item answered “yes” or “no”; a “yes” response scores 1 point and a “no” response scores 0 points. A GOAL score ≥2 indicates a high risk of OSA [17].
6. Berlin score consists of eleven items divided into three categories: severity of snoring; daytime sleepiness; high blood pressure or obesity. If two or more of the three categories are positive, the individual is considered to be at high risk of having OSA [12].

### 2.4 Respiratory polygraphy

2.5 Respiratory PG was performed using ApneaLink™ Plus devices (ResMed R&D, Germany). This type III portable recorder measures airflow through a nasal pressure sensor, respiratory effort (thoracic movement), and pulse oximetry (Nonin provided by ResMed). Participants were provided with a PG device that was set up in their home between 8pm and 10pm by one of the four trained research assistants, who returned the next morning to collect the device. All PG data were transformed into European Data Format (EDF) files to allow reading in Noxturnal software (version 6.2, Nox Medical, Reykjavik, Iceland). The sleep recordings were then manually scored by a certified sleep physician (APW) with more than 10 years of experience in respiratory sleep medicine who was unaware of the participant's data. Only PG data with ≥4 h of recording with airflow, pulse oximetry and respiratory effort signal were considered valid and included in the analysis. Respiratory events were scored according to the 2012 American Academy of Sleep Medicine (AASM) manual [19]. The apnoea-hypopnoea index (AHI) was calculated as the sum of all apnoeas and hypopnoeas divided by the estimated sleep time based on participant's report of lights off to lights on. The severity of OSA was classified as follows: mild (AHI 5 to <15/h), moderate (AHI 15 to <30/h) or severe (AHI ≥30/h). For the primary analyses, clinically relevant OSA was defined as AHI≥15 events/hour (i.e., moderate-to-severe OSA), as this threshold is widely accepted in clinical practice to guide further evaluation or treatment and is more appropriate for population-based risk stratification than restricting analyses to severe OSA alone. As the ventilatory device used records airflow, oximetry, and a single abdominal respiratory effort signal, we were unable to reliably classify central events; consequently, our analyses focused on OSA as defined by the available signals. **Statistical analysis**.

Continuous variables are presented as median values with interquartile range (IQR) or mean with standard deviation (SD), and categorical variables are reported as proportions (%). Between-group comparisons were performed using the Chi-squared test, Student's unpaired *t*-test, or Wilcoxon's rank-sum test, as appropriate.

Correlations between questionnaire scores and the AHI from PG recordings were analysed using Pearson coefficient for normally distributed data, and Spearman coefficient for non-normal distributed data. In addition, we performed a best cut-off analysis for each of the questionnaires using the Youden point method [20], in order to derive standardized and comparable thresholds across instruments for descriptive and comparative purposes, rather than to establish definitive clinical decision points.

The following performance values were calculated for each OSA screening questionnaire compared with PG-based AHI (≥5/h, ≥15/h or ≥30/h): sensitivity, specificity, PPV, negative predictive value (NPV), area under the receiving operating characteristic curve (AUC). AUC values were compared between scales using the between-area correlation method [21].

Statistical analyses were performed with Stata (StataCorp, College Station, Texas, USA) and Medcalc (MedCalc Software Ltd Acacialaan, Ostende, Belgium), and a two-tailed p-value of <0.05 was considered statistically significant.

## 3 Results

### 3.1 Description of the sample

Overall, 1810 subjects with valid PG data and completed questionnaires were included in the analysis. The study population was predominantly female (64.3%) and young (age <60 years in 81.2%) (Table 1). Mild OSA was present in 31.6% of the study population, 8.9% had moderate OSA, and 2.7% had severe OSA; median age and median neck circumference increased with OSA severity (Table 1).

**Table 1:** Clinical characteristics of participants with valid polygraphy data and complete questionnaires recruited in the BeSAS study (n = 1810).

	All^a^	No OSA	Mild OSA	Moderate OSA	Severe OSA	p-value^b^
Number of cases, n (%)	1810 (100%)	1029 (56.9%)	572 (31.6%)	161 (8.9%)	48 (2.7%)	
Male sex, n (%)	647 (35.7%)	315 (30.6%)	238 (41.6%)	71 (44.1%)	23 (47.9%)	<0.001
Area of residence, n (%)						
Rural	955 (52.8%)	604 (58.7%)	273 (47.7%)	67 (41.6%)	11 (22.9%)	<0.001
Urban	855 (47.2%)	425 (41.3%)	299 (52.3%)	94 (58.4%)	37 (77.1%)	
Age, years	43 (34-55)	39 (32-50)	46 (37-58)	54 (43-64)	55 (49-64)	<0.001
Age group, n (%)						<0.001
<40 years	767 (42.4%)	548 (53.3%)	181 (31.6%)	34 (21.1%)	4 (8.3%)	
40-59 years	702 (38.8%)	335 (32.6%)	273 (47.7%)	69 (42.9%)	25 (52.1%)	
60 years	341 (18.8%)	146 (14.2%)	118 (20.6%)	58 (36.0%)	19 (39.6%)	
BMI, kg/m^2^	23.6 (20.5-27.8)	22.3 (19.7-25.7)	25.3 (21.9-29.6)	26.4 (22.6-30.8)	31.6 (27.2-37.8)	<0.001
BMI category, n (%)						<0.001
Normal	1080 (59.7%)	721 (70.1%)	282 (49.3%)	67 (41.6%)	10 (20.8%)	
Overweight	420 (23.2%)	213 (20.7%)	154 (26.9%)	47 (29.2%)	6 (12.5%)	
Obese	310 (17.1%)	95 (9.2%)	136 (23.8%)	47 (29.2%)	32 (66.7%)	
Neck circumference, cm	34.0 (32.0-37.0)	34.0 (32.0-36.0)	35.0 (33.0-38.0)	36.0 (34.0-38.5)	39 (36.5-41.0)	<0.001
Abdominal obesity, n (%)	673 (37.2%)	300 (29.2%)	255 (44.6%)	82 (50.9%)	36 (75.0%)	<0.001
Alcohol use, n(%)	200 (11.1%)	91 (8.8%)	80 (14.0%)	19 (11.8%)	10 (20.8%)	0.002
Current smoking, n (%)	146 (8.1%)	71 (6.9%)	53 (9.3%)	16 (9.9%)	6 (12.5%)	0.137
Snoring, n (%)	492 (27.2%)	200 (19.4%)	186 (32.5%)	69 (42.9%)	37 (77.1%)	<0.001
Hypertension, n (%)	836 (46.2%)	392 (38.1%)	302 (52.8%)	104 (64.6%)	38 (79.2%)	<0.001
Diabetes, n (%)	74 (4.2%)	26 (2.6%)	32 (5.8%)	12 (7.8%)	4 (8.7%)	<0.001
Reported sleep time^c^, h	7.2 ± 1.3	7.1 ± 1.2	7.0 ± 1.4	6.8 ± 1.5	7.0 ± 1.2	0.38
AHI, events/h	4.2 (2.0-8.1)	2.1 (1.2-3.3)	7.4 (6.1-9.9)	18.6 (16.2-21.7)	36.9 (32.2-50.8)	<0.001

∗AHI, apnoea-hypopnoea index; BMI, body mass index; ESS, Epworth Sleepiness Scale; NoSAS, Neck-Obesity-Snoring-Age-Sex score; OSA, obstructive sleep apnoea; kg/m^2^, kilograms per square meter; cm, centimetre; n, number; (%), percentage, h, hour. Data are presented as median (interquartile range), or number of participants (%).

a n = 1810 for all variables except for neck circumference (n = 1805) and diabetes (n = 1770).

b Data were analysed using Pearson Chi-squared test or Fischer exact text for categorical variables and Kruskal-Wallis or ANOVA tests for continuous variables; p-value are for comparison between OSA severity subgroups.

c Sleep time was estimated based on light off-light on times reported by the participants.

### 3.2 Optimal cut-off scores, and correlation with AHI

The optimal cut-off scores in our sample to dichotomize participants into groups having low risk versus high risk of mild-to-severe OSA were similar to the current reference values for all questionnaires except for NoSAS, for which the optimal cut-off value was 5 (versus reference score of 8) (Table 2). The NoSAS score showed the best correlation with AHI values (coefficient 0.434, p < 0.001), followed by GOAL (0.415, p < 0.001), STOP-Bang (0.389, p < 0.001) and No-Apnea (0.385, p < 0.001).

**Table 2:** Analysis of best cut-off value for six obstructive sleep apnoea screening questionnaire based on the Youden point method (n = 1810).

	Reference cut-off	Mild OSA	Moderate OSA	Severe OSA	Mean best cut-off
NoSAS	8	3.5	5.5	5.5	5
Berlin	2	0.5	0.5	1.5	2
STOP	2	1.5	1.5	1.5	2
STOP-Bang	3	2.5	2.5	2.5	3
GOAL score	2	1.5	1.5	1.5	2
No-Apnea	3	1.5	2.5	3.5	3

∗OSA, obstructive sleep apnoea; NoSAS, Neck circumference-Obesity-Snoring-Age-Sex score; STOP, Snoring-Tiredne-Observed apnoeas-High blood Pressure score; STOP-Bang, Snoring-Tiredness-Observed apnoeas-High blood Pressure-BMI-Age-Neck circumference-Gender score; GOAL, Gender-Obesity-Age-Loud snoring.

### 3.3 Score performances

**Fig. 1:**
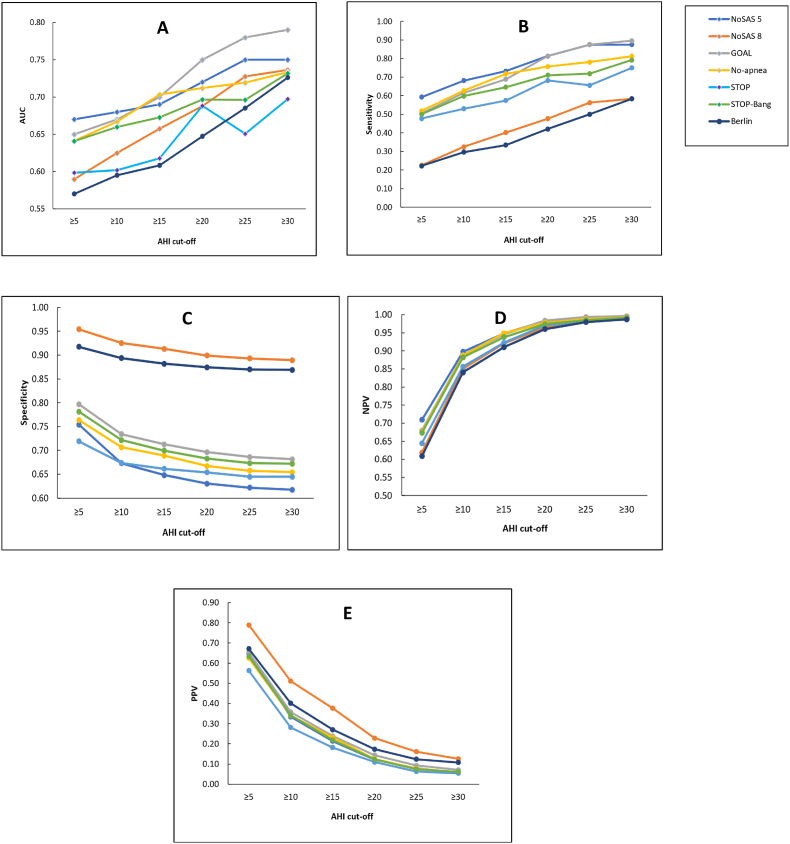
Performance of six obstructive sleep apnoea screening scores (NoSAS, GOAL, No-Apnea, STOP, STOP-Bang, Berlin) at different AHI cut-off values (5,10,15,20,25,30). A. Area under the receiver operator characteristic curve (AUC); B. Sensitivity value; C. Specificity value; D. Negative predictive value; E. Positive predictive value (PPV).

Table 3 and Fig. 1 detail the performance parameters for each score compared with the AHI, including at different AHI cut-off values. At an AHI cut-off of 15/h, the highest AUC value was seen for No-Apnea and GOAL (0.70), followed by NoSAS_5_ (0.69), STOP-Bang (0.67), NoSAS_8_ (0.66), STOP (0.62) and Berlin (0.61). At an AHI cut-off of 15/h, NOSAS_8_ had the highest specificity (0.91), followed by Berlin (0.88), GOAL (0.71) and STOP-Bang (0.70). The PPV at the same AHI cut-off value was low for all scores, being 0.38, 0.27, 0.24, and 0.23 for NoSAS_8_, Berlin, GOAL, and No-Apnea, respectively. NPV values were high for all scores (0.91–1.00), and similar at AHI cut offs at 15/h and 30/h. Comparisons between, AUCs showed that differences between questionnaires were modest, and several were not statistically significant (p > 0.05).

**Table 3:** Performance values for the different obstructive sleep apnoea screening questionnaires compared with polygraphy (n = 1810).

	Sensitivity (95% CI)	Specificity (95% CI)	PPV (95% CI)	NPV (95% CI)	AUC (95% CI)
AHI ≥5 events/hour					
NoSAS_8_	0.23 (0.20-0.25)	0.95 (0.94-0.97)	0.79 (0.74-0.84)	0.62 (0.59-0.64)	0.59 (0.55-0.63)
NoSAS_5_	0.59 (0.56-0.63)	0.75 (0.73-0.78)	0.65 (0.62-0.67)	0.71 (0.69-0.73)	0.67 (0.65-0.70)
No-apnea	0.52 (0.48-0.55)	0.76 (0.74-0.79)	0.63 (0.59-0.66)	0.68 (0.65-0.70)	0.64 (0.62-0.67)
Berlin	0.22 (0.19-0.25)	0.92 (0.90-0.93)	0.67 (0.61-0.73)	0.61 (0.58-0.63)	0.57 (0.53-0.61)
STOP	0.48 (0.44-0.51)	0.72 (0.69-0.75)	0.56 (0.53-0.60)	0.64 (0.62-0.67)	0.60 (0.57-0.63)
STOP-Bang	0.50 (0.47-0.54)	0.78 (0.76-0.81)	0.63 (0.60-0.67)	0.67 (0.65-0.70)	0.64 (0.62-0.67)
GOAL	0.51 (0.47-0.54)	0.80 (0.77-0.82)	0.65 (0.62-0.68)	0.68 (0.66-0.70)	0.65 (0.62-0.68)
**AHI ≥15 events/hour**					
NoSAS_8_	0.40 (0.34-0.47)	0.91 (0.90-0.93)	0.38 (0.31-0.44)	0.92 (0.91-0.93)	0.66 (0.62-0.69)
NoSAS_5_	0.73 (0.67-0.79)	0.65 (0.62-0.67)	0.21 (0.20-0.23)	0.95 (0.94-0.96)	0.69 (0.65-0.73)
No-apnea	0.72 (0.66-0.78)	0.69 (0.67-0.71)	0.23 (0.20-0.26)	0.95 (0.94-0.96)	0.70 (0.68-0.73)
Berlin	0.33 (0.27-0.40)	0.88 (0.87-0.90)	0.27 (0.22-0.32)	0.91 (0.90-0.92)	0.61 (0.57-0.64)
STOP	0.57 (0.51-0.64)	0.66 (0.64-0.68)	0.18 (0.15-0.21)	0.92 (0.91-0.94)	0.62 (0.59-0.64)
STOP-Bang	0.65 (0.58-0.71)	0.70 (0.68-0.72)	0.22 (0.19-0.25)	0.94 (0.92-0.95)	0.67 (0.65-0.70)
GOAL	0.69 (0.62-0.75)	0.71 (0.69-0.73)	0.24 (0.22-0.26)	0.95 (0.93-0.96)	0.70 (0.67-0.73)
**AHI ≥30 events/hour**					
NoSAS_8_	0.58 (0.44-0.72)	0.89 (0.87-0.90)	0.13 (0.08-0.17)	0.99 (0.98-0.99)	0.74 (0.71-0.77)
NoSAS_5_	0.87 (0.75-0.95)	0.62 (0.59-0.64)	0.06 (0.05-0.07)	0.99 (0.99-1.00)	0.75 (0.67-0.83)
No-apnea	0.81 (0.70-0.92)	0.65 (0.63-0.68)	0.06 (0.04-0.08)	0.99 (0.99-1.00)	0.73 (0.71-0.76)
Berlin	0.58 (0.44-0.72)	0.87 (0.85-0.88)	0.11 (0.07-0.15)	0.99 (0.98-0.99)	0.73 (0.70-0.76)
STOP	0.75 (0.63-0.87)	0.64 (0.62-0.67)	0.05 (0.04-0.07)	0.99 (0.98-1.00)	0.70 (0.67-0.72)
STOP-Bang	0.79 (0.68-0.91)	0.67 (0.65-0.69)	0.06 (0.04-0.08)	0.99 (0.99-1.00)	0.73 (0.71-0.76)
GOAL	0.90 (0.77-0.97)	0.68 (0.66-0.70)	0.07 (0.06-0.08)	1.00 (0.99-1.00)	0.79 (0.74-0.84)

∗AHI, apnoea-hypopnoea index; AUC, area under the receiver operator characteristic curve; CI, confidence interval; NPV, negative predictive value; PPV, positive predictive value, OSA, obstructive sleep apnoea; NoSAS, Neck circumference-Obesity-Snoring-Age-Sex score; STOP, Snoring-Tiredness-Observed apnoeas-High blood Pressure score; STOP-Bang, Snoring-Tiredness-Observed apnoeas-High blood Pressure-BMI-Age-Neck circumference-Gender score; GOAL, Gender-Obesity-Age-Loud snoring.

## 4 Discussion

To the best of our knowledge, this is the first study evaluating the performances of different OSA prediction scores in an African general population. The three scores that showed the highest AUC at each AHI cut-off used to define OSA were GOAL, No-Apnea and NoSAS_5_. To detect moderate-to-severe OSA, NoSAS_8_ had the highest specificity, and PPV.

One of the most widely used OSA screening tools is the STOP-Bang questionnaire [22]. While this tool seems to perform well for OSA screening in individuals from Western countries [23,24], it performed poorly compared with NoSAS_5_, GOAL and No-Apnea in our African sample. Pivetta et al. recently conducted a meta-analysis on the use of STOP-Bang across different geographic regions in clinical settings [22], They showed that STOP-Bang performance to detect moderate-to-severe OSA was high in North America, Europe and Middle East (AUC 0.89, 95% confidence interval [CI] 0.85–0.91), but lower in East Asia (AUC 0.52, 95% CI 0.48–0.56) and South or Southeast Asia (AUC 0.70, 95% CI 0.66–0.74) [22]. The AUC in South or Southeast Asian populations is similar to that found in African individuals in our study (0.67, 95% CI 0.65–0.70), although populations were different (clinical population in the Pivetta study versus a general population in our study).

Variations in STOP-Bang performances between different geographic groups have been explained by differences in obesity between populations and by some specific craniofacial features among ethnic groups [25,26]. Available data suggest that OSA populations in Asia primarily display features of cranio-facial skeletal restriction that are not reflected by BMI. African American individuals have more obesity and enlarged upper airway soft tissues, while Caucasian individuals show evidence of both bony and soft tissue [25,26]. Unfortunately, these comparisons of OSA prevalence and STOP Bang questionnaire performances across ethnic groups did not include populations living on the African continent due to data unavailability. Our study provides data on a large sample in West Africa for the first time, but further studies are needed to evaluate the performance of the STOP-Bang questionnaire in populations living in other West African countries and other African regions to study cranio-facial and obesity patterns involved in OSA among different African populations.

Another widely-used scale is the NoSAS score, primarily designed in a Caucasian general population (HypnoLaus), and validated in a Brazilian ethnically mixed population (Episono) with a suggested cut-off of 8 points for positivity [15]. This tool has been further validated in various specific clinical populations with overall good performances [[27], [28], [29], [30], [31], [32]], except in non-obese women for whom false negative results have been reported [33]. It is interesting to note that the optimal score cut-off values in our sample were similar to the established reference cut-off values for all the scores analysed in the current study except for NoSAS, for which the optimal cut-off was 5 (compared to the reference cut-off of 8). Bauters et al. recently showed that the best cut-off value for NoSAS in women should be 6 instead of 8 [33]. Therefore, the high proportion of women in our sample (64.3%) could be one explanation for the difference in NoSAS cut-off between our study and the current reference value.

The practical simplicity of certain instruments is an important consideration for screening in low-resource or community settings. For example, the No-Apnea score includes only two objectively measured variables (age and neck circumference), and the GOAL questionnaire comprises four easily obtained items (gender, obesity, age, and snoring), making both tools particularly feasible where time, training, or equipment are limited. Evidence from comparative studies suggests that simpler scores may perform similarly to more complex instruments in identifying individuals at risk for OSA. In a clinical study involving patients with cerebral infarction, comparing No-Apnea with multiple other questionnaires including NoSAS and STOP-Bang, discrimination between instruments at clinically relevant AHI thresholds (AHI: >5–15/h mild, >15–30/h moderate, and >30/h severe) was broadly comparable, indicating that simpler models can provide useful stratification despite fewer variables [34]. Additionally, the STOP questionnaire, a concise four-item tool developed for easy application in preoperative settings, demonstrated high sensitivity for detecting moderate to severe OSA, illustrating how brief, focused instruments can be effective in identifying individual at elevated risk [14]. In a cohort of psychiatric patients, where OSA screening is particularly challenging due to overlapping symptoms and adherence issues, the No-Apnea questionnaire achieved the highest sensitivity for detecting any OSA (96%, AUC 0.68), followed by GOAL (92%, AUC 0.59). However, specificities were low across instruments, underscoring the need for confirmatory diagnostic testing [35]. Similarly, in elderly patients, the GOAL questionnaire demonstrated high sensitivity at AHI cut-offs of 5 and 15 events/hour compared with other commonly used instruments, but relatively low specificities [36].

However, in our study, neither No-Apnea nor GOAL demonstrated clearly superior or clinically decisive performance compared with the other instruments evaluated. Although their simplicity supports feasibility, their discriminatory ability remained modest, reinforcing that ease of administration alone does not guarantee adequate predictive performance.

In countries with easy access to sleep recording, NPV is probably the most important performance characteristic for screening scores because they are mainly used to exclude significant OSA in individuals with low or intermediate clinical probability. In LMICs with few or no sleep recording facilities, a score should have a good PPV to be useful. This allows detection of probable OSA or selection of only high-risk individuals for the few available sleep examination slots. Although regions without diagnostic equipment are unlikely to have the means to treat patients with positive pressure therapy, a positive diagnosis of OSA based on high PPV in people with comorbidities (obesity, cardiovascular, neuro-cognitive) and/or sleep apnoea symptoms may provide a good incentive to promote low-cost treatments with proven efficacy in OSA (including physical activity, obesity management, myofunctional therapy, positional therapy, and thermoformed mandibular advancement devices) [37]. Unfortunately, at an AHI cut-off of ≥15/h, a threshold often considered as a clinically relevant demarcation for OSA, all the scores evaluated showed a PPV below 0.40, with NoSAS_8_ having the highest PPV (0.38).

These low PPV values reflect the fact that there is currently no suitable score for clinical and therapeutic decision making in resource-limited settings where access to sleep recordings remains very limited [38]. This situation calls for two actions. The first concerns the need for health authorities to invest in sleep medicine to make diagnostic tools available because sleep apnoea is as common in LMICs as it is in developed countries [18]. The second is the need to accelerate clinical research to develop sufficiently efficient scores that could one day enable PG- or PSG-free diagnosis of sleep apnoea, which would be very beneficial and even essential for countries with limited resources. For now, the NoSAS_8_ score that showed the highest specificity in the studied population, in conjunction with presence of OSA symptoms, could be useful to manage individuals in the Beninese population.

The strengths of this study include the large sample size, and an objective evaluation of OSA. Furthermore, this is the first study assessing the performances of various OSA screening scores in an African population. However, this study has some limitations that need to be acknowledged. First, we used PG instead of the gold standard PSG to diagnose OSA. Nevertheless, PG is a well-validated tool that is in widespread use for OSA diagnosis worldwide [39]. Second, PG could not be performed the same day as the questionnaires were administered, but the gap between these assessments was less than one month, and given that questions within each score reflect a general situation (e.g. do you snore?), we do not expect that day-to-day variation would have influenced the study findings. Third, participants were recruited at the household level, and intra-household correlation could not be formally accounted for. Finally, diagnostic performance metrics were estimated without sampling weights, and analyses were not reweighted for sex distribution; therefore, results should be interpreted as reflecting intrinsic test performance rather than population-representative estimates.

In summary, this study provides the first comparison of the performance of various screening scores for OSA compared with PG in an African general population. GOAL, No-Apnea and NoSAS_5_ showed the highest general performance as measured by the AUC at different AHI thresholds. Although overall performance was modest for all instruments, NoSAS_8_ showed the highest PPV among the evaluated scores. NoSAS_8_ might therefore be a useful support for OSA diagnosis in resource-constrained settings where PG or PSG are not accessible for objective assessments. Further studies are needed to develop PG-free diagnostic tools for OSA.

## CRediT authorship contribution statement

**Ablo Prudence Wachinou:** Writing – review & editing, Writing – original draft, Project administration, Investigation, Funding acquisition, Formal analysis, Data curation, Conceptualization. **Boubacar Djelo Diallo:** Writing – original draft, Data curation. **Terence Totah:** Investigation, Formal analysis, Data curation. **Geoffroy Solelhac:** Writing – original draft, Data curation. **Mathieu Berger:** Writing – original draft, Data curation. **Pervenche Fosto:** Writing – review & editing, Writing – original draft, Formal analysis, Data curation. **Salmane Amidou:** Writing – review & editing, Investigation, Data curation, Conceptualization. **Attanon Arnauld Fiogbe:** Writing – review & editing, Investigation, Data curation. **Pedro Marques-Vidal:** Writing – review & editing, Writing – original draft, Data curation. **Gildas Agodokpessi:** Writing – original draft, Validation, Investigation, Formal analysis, Data curation. **Dismand Houinato:** Writing – review & editing, Investigation, Conceptualization. **Roch Christian Johnson:** Writing – original draft, Data curation. **Pierre-Marie Preux:** Writing – review & editing, Formal analysis, Data curation. **Raphael Heinzer:** Writing – review & editing, Writing – original draft, Validation, Funding acquisition, Data curation, Conceptualization.

## Funding support and role of the sponsor

The study was funded by the “Ligue Pulmonaire Vaudoise, Lausanne, Switzerland”, a non-governmental organisation dedicated to pulmonary health. The funder had no role in study design, data collection, data analysis, data interpretation, or writing of the report. All authors had full access to study database and the corresponding author had final responsibility for the decision to submit for publication.

## Declaration of competing interest

The authors declare that they have no known competing financial interests or personal relationships that could have appeared to influence the work reported in this paper.

## Glossary

1. **Obstructive Sleep Apnoea (OSA)**: A sleep disorder characterized by repeated episodes of complete or partial obstruction of the upper airway during sleep, leading to reduced or paused breathing
2. **Apnoea-Hypopnoea Index (AHI)**: A measure that quantifies the severity of sleep apnoea. It is calculated as the number of apnoea and hypopnoea events per hour of sleep.
3. **Polygraphy (PG)**: A type III diagnostic device used to measure respiratory parameters like airflow, respiratory effort, and oxygen saturation during sleep.
4. **NoSAS Score**: A screening tool for OSA based on five criteria: neck circumference, BMI, snoring, age, and sex
5. **STOP-Bang**: A screening questionnaire combining the STOP criteria (snoring, tiredness, observed apnoeas, high blood pressure) with BMI, age, neck circumference, and gender
6. **GOAL Score**: A screening tool assessing gender, obesity, age, and loud snoring to predict the risk of OSA
7. **Positive Predictive Value (PPV)**: The proportion of true positives among those identified as positive by a screening tool
8. **Sensitivity**: The ability of a test to correctly identify individuals with OSA
9. **Specificity**: The ability of a test to correctly identify individuals without OSA
